# Anti-biofilm Activity of a Lytic Phage Against Vancomycin-Resistant *Enterococcus faecalis*

**DOI:** 10.30699/IJP.2022.541855.2760

**Published:** 2022-08-12

**Authors:** Forough Goodarzi, Masoumeh Hallajzadeh, Mohammad Sholeh, Malihe Talebi, Vahid Pirhajati Mahabadi, Nour Amirmozafari

**Affiliations:** 1 *Department of Microbiology, School of Medicine, Iran University of Medical Sciences, Tehran, Iran*; 2 *Neuroscience Research Center, Iran University of Medical Sciences, Tehran, Iran*; 3 *Cellular and Molecular Research Center, Iran University of Medical Sciences, Tehran, Iran*

**Keywords:** Bacteriophages, Biofilms, Enterococcus faecalis, PCR, Phage Therapy, Vancomycin Resistance

## Abstract

**Background & Objective:**

This study aims to isolate a lytic bacteriophage against planktonic *Enterococcus faecalis* V583 culture and evaluate its ability to disrupt and inhibit biofilm.

**Methods:**

An anti-*E. faecalis* phage was isolated from sewage and visualized by electron microscopy, the vB_EfsS_V583 (V583) host range was determined by spot test on 13 *E. faecalis *clinical strains. Inhibition and degradation experiments were designed to investigate the effect of phage on biofilm. In the inhibition and degradation assay, biofilms were formed in the presence and absence of phage, respectively. Finally, crystal violet method tested the effect of phage on biofilm.

**Results:**

Phage V583 belongs to the *Siphoviridae* family and can infect all *E. faecalis *strains. Antibacterial activity has been shown to degrade and inhibit biofilm produced by V583. The study results showed that phage v583 is more efficient in biofilm inhibition than biofilm degradation. In both assays, phage-treated wells' absorption is less than untreated wells. These results were confirmed by Colony-forming unit reduction in the treated biofilm.

**Conclusion:**

The anti-biofilm activity showed that phage therapy using phage V583 might be an alternative tool to remove *E. faecalis* biofilms.

## Introduction

Increasing levels of antibiotic resistance threaten human health, which is widely known as a global emergency and therefore requires urgent attention (1). In 2013, the Centers for Disease Control and Prevention (CDC) classified the antibacterial resistance threats and declared VRE enterococci to be a serious threat to deal with today (2). Enterococci are important nosocomial pathogens that can survive for a long time on medical equipment. Due to their high tolerance and genetic plasticity, they are spread widely in the hospital environment (3). The National Institute of Health estimated that biofilms account for approximately 80% of human bacterial infections (4). Biofilm is a microbial population of cells attached irreversibly to a surface, i.e., a wide variety of medical devices, living tissues encased in extracellular polymeric substances (EPSs) consisting of proteins, extracellular DNA, and polysaccharides (5). Bacteria in a biofilm display various biological properties compared to their planktonic state (6). A characteristic property of the mature biofilm is that it has antibiotic resistance. Moreover, biofilm bacteria become inaccessible to immune cell attacks (7). The matrix (EPSs) formulates structural stability and protection for the biofilm (8). It is responsible for limited penetration of substances and binds antimicrobials; as a result, it will decrease the concentration of the antibiotic entering the biofilm and provide effective resistance for biofilm; thus, due to the chronic recurrent infections, patient morbidity and biofilms mortality have great significance for public health (9). *Enterococcus faecalis* is well-known as an opportunistic pathogen inhabiting the gastrointestinal tract. It is responsible for 80–90 % of all enterococcal infections, such as endocarditis, bacteremia, urinary tract, burn, chronic wound infection, and meningitis (10). It appears mainly as a nosocomial and community-acquired infection due to its ability to form mature biofilms that are extremely difficult to treat (11). *E. faecalis *is a bacterial species most commonly isolated from biofilms (an important virulence factor in the pathogenesis). Due to frequent fractures of antibiotic therapy, developing new strategies for fighting biofilms is currently one of medicine's major problems (12). In this context, taking advantage of bacteriophages might become a new anti-biofilm strategy for inhibiting biofilm formation and contributing to the destruction of biofilms (13). Therapeutic use of phages as bacteria-eliminating factors has many advantages that are discussed in detail as follows: Phage isolation is easy, fast, relatively inexpensive, and highly strain-specific, it does not infect normal flora and is safe for human cells, phages have bactericidal activity against antibiotic-resistant strains and biofilm, and the process of phage-resistant bacteria is ten times slower (14). Phages produce large amounts of depolymerization that, by destroying the EPS, facilitates the penetration of phages into the biofilm's interior layers. Thus, crucial receptors are available for the initiation of productive phage infection (15). The provided studies revealed the effects of phages on bacterial biofilms to be even more successful in some cases in comparison to the antibiotic treatment (16, 17). Only a few studies have explored any effects of phage therapy in the treatment of *E. faecalis* biofilm in the last few decades. In this study, we decided to work with VRE (ATCC 700802) since it is a clinically important multidrug-resistant pathogen. A lytic *E. faecalis*-infecting phage was isolated from sewage and characterized. We demonstrated its efficacy to disrupt (removing an existing biofilm) and inhibit (blocking the onset of biofilm development) *E. faecalis *V583 biofilms in vitro and planktonic clinical strains.

## Material and Methods

**Isolation and Identification of Vancomycin Resistance Enterococci**

Forty-six isolates of Vancomycin-resistant enterococci were collected from various clinical samples of Shariati Hospital microbiology laboratory (Tehran, Iran). Enterococcal isolates were initially re-identified on a series of microbiological tests (catalase, growth in bile-esculin and BHI agar medium containing 6.5% salt, PYR assay, motility assay, and arabinose fermentation) (18). Brain heart infusion broth (Merck, Germany) or agar was used to grow bacterial strains at 37°C under aerobic conditions. 

**Antimicrobial Susceptibility Testing**

The minimum inhibitory concentration (MIC) assay was determined using E-test (Liofilchem, Roseto Degli Abruzzi, Italy) strips for vancomycin based on CLSI 2017 guideline (19). *E. faecalis* ATCC29212 and *Staphylococcus aureus* ATCC25923 were used as controls.

**Multiplex Polymerase Chain Reaction**

The multiplex PCR assays were performed to determine the vancomycin resistance gene (*VanA*) and confirm the identification of the isolate as *E. faecalis *(20). (Table 1). The PCR program used in this study was as follows: initial denaturation at 94°C for 5 min, 30 cycles of denaturation at 94°C for 1 min, annealing at 54°C for 1 min, extension at 72°C for 1 min, and a final extension at 72°C for 10 min.

**Table 1:** The Primer Sequences used in the multiplex PCR assay

Gene	Primer Sequence	Product size	Reference
*E. faecalis*	TCTGATTGACATGAACTA-5′ GTCAATCGAAACTTAGCA-5′	941	**(** 20 **)**
*vanA*	ATAACGTTGAAAATAAGATAAGTA-5′ AACTAGCATAATCGCAATTTCCCC-5′	1030	**(** 20 **)**

**Isolation of Bacteriophage**

Isolation of phage was performed using the previously described method with slight modifications (21). The *E. faecalis *V583 was used as a host for phage isolation and propagation. Briefly, hospital sewage effluent from Shariati Hospital (Tehran, Iran) was centrifuged (centrifuge 5430R, rotor FA-45-24-11HS; Eppendorf) for 15 min at 4,000 rpm to precipitate bacteria. The supernatant was filtered for the presence of phages through a sterile membrane filter 0.45 μm -pore-size (Merck Millipore, Ltd., Ireland) and stored at 4°C. 10 mL of logarithmic V583 cultures were inoculated with 10 mL of filtered sewage and incubated at 37°C, 80 rpm for 24 h. This enrichment process was repeated several times until complete lysis was obtained. 

**Plaque Assay, Purification, and Titration**

The double-layer agar (DLA) method was performed for phage plaque assay (23). 1 mL of phage lysates was filtered and was added to 5 mL melted BHI soft agar (0.7% wt/vol agar, 7 mL, 45°C) containing 0.5 mL of logarithmic V583 culture. The mixture was spread on BHI agar and then incubated overnight at 37°C as described above. A clear plaque was transferred into a tube containing BHI broth and logarithmic phase V583 for purifying phage particles, and plaque assay was repeated three times. The concentration of phage particles within the lysate was determined according to the standard method (23). Briefly, for titration of phage particles, 100 µL of the lysates were serially diluted 10-fold into 900 µL of BHI broth. 100 µL of the logarithmic phase of V583 and each phage dilution were added to 3 mL of BHI soft agar, and the plaques were obtained as described previously. Finally, the plaques were counted and expressed as plaque-forming units (PFU/mL).

**Electron Microscopy**

The morphology of the isolated phage particles was examined by Transmission Electron Microscopy (TEM), as previously described (22). Briefly, 10 µL of purified phage particles were deposited onto the surface of a carbon-coated copper grid for 3-5 min and stained with 1% (w/v) uranyl acetate (pH = 7). Micrographs were obtained using a Zeiss LEO 906 TEM (Carl Zeiss LEO EM 906 E, Germany) at an accelerating voltage of 100 kV.

**Host Range Determination**

 The host range was determined by Kutter's spot test method on 13 clinical strains of vancomycin-resistant *Enterococcus faecalis* (VREfs) (23). For this purpose, bacterial strains were first cultured in a liquid BHI medium at 37°C for 24 h. Then, 1 mL of each strain was added to 6 mL of BHI soft agar and spread onto BHI agar. After getting dried, 10 µL of the pure phage suspension (10^9^ PFU/mL) was spotted on the bacterial strain. Following overnight incubation at 37°C, lytic activity was checked for bacterial susceptibility to the phage. The double-layer agar method was also performed on the condition that the spot test was positive. Thirty-four clinical strains of *Enterococcus faecium* resistant to vancomycin, JH-2-2 (*E. faecalis* sensitive vancomycin), and *Enterococcus gallinarum* were also utilized to determine the host range.

**Inhibitory Effect of Phage on Biofilms**

The Anti-biofilm effect of phage was performed as described in the previous publication (24). To investigate the inhibitory effect of phage on biofilm formation, 1 to 7-day-old biofilm was formed in the presence of phage. First, Single colonies of *E. faecalis* strain V583 were cultured at 37°C for 24 h. After the incubation, 1 mL of overnight bacteria was added to 9 mL of BHI broth medium and incubated for 2 h with 150 rpm shaking at 37˚C to reach OD ~ 0.5. For instance, to evaluate the inhibitory effect, 20 μL of OD ~0.5 bacteria were inoculated to 220 μL of BHI broth and 10 μL of phage lysate (10^9^ PFU/mL) in 96-well plates (Biofil: TCP001096). By the time of quantifying inhibition using CV staining, the medium and planktonic cells were aspirated in each well, and fresh 240 μL of BHI media and 10 μL phage were added every 24 h. After incubation (1 to 7 days), the suspension was drained from wells and rinsed with sterile distilled water three times. The biofilm formation was fixed with 200 μL of methanol for 20 min, followed in turn by methanol aspiration and air drying, staining with 200 μL of 1% crystal violet (Sigma, C0775) for 20 min, washing three times with distilled water to remove excess stain from the wells, dissolving CV stain in biofilm using 200 μL of 96-degree ethanol, and measuring optical absorption of CV stain at 630 nm by a plate reader (Biotech, Synergy2-Cam4, Software-Gen5-1.08). BHI broth containing ATCC 700802 without phage was used as a positive control. The biofilm formation was performed in triplicate for treated and untreated samples.

**Disruption Effect of Phage on Biofilms**

 For disruption assay, 1 to 7-day-old biofilms were formed in the absence of phage and then treated. 20 μL of OD~0.5 V583 and 230 μL of BHI broth were added to microtiter plates. After biofilms were established for 1 to 7 days, biofilms were treated with 10 μL of the lysate (10^9^ PFU/mL) for 24 h at 37˚C. Absorption of CV stain in phage-treated biofilms was quantified as described before. BHI broth containing ATCC 700802 without phage was used as a positive control. Biofilm formation was performed in triplicate for treated and untreated samples.

**Colony-forming Unit Assay**

In both inhibition and disruption experiments, parallel cultures were also utilized to determine live cells in biofilm by measuring colony-forming units, as described previously (24). After incubation of biofilms with phage, the suspension was aspirated, and wells were washed with sterile distilled water. Cells within the biofilm were scratched with a sterile taper device and inoculated in a series of 10-fold dilutions into 100 μL of normal saline. The solutions were cultured on BHI agar using sterile Pasteur Pipette and incubated in a 37°C incubator for 24 h.

**Statistical Analysis**

Data were assessed and analyzed using GraphPad Prism 5.0(GraphPad Software, Inc., San Diego, CA, USA) software. The students' t-test evaluated the effect of phage treatment on biofilm and the results were significant when the p-value became less than 0.05. The error bars in the figures represent the mean, and standard deviation (SD). Each experiment was replicated three times**.**

## Results

**Identification of Vancomycin Resistance** ***E. faecalis***

**Fig. 1:**
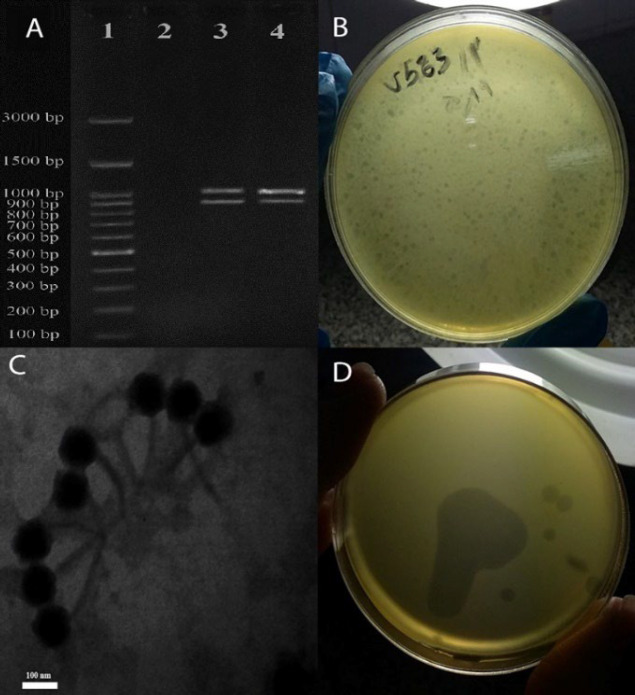
Multiplex PCR assay with two primer sets for identifying *E. faecalis* strains and detection of vanA gene. Lane 1: 100-bp DNA ladder (SMO Bio), Lane 2: Negative control, Lane 3: positive control, Lane 4: Clinical strains **(A)**. Morphology of phage V583 plaques by DLA method **(B)**. Micrograph of phage V583 by transmission electron micrographs (TEM). The scale bar represents 100 nm **(C)**. Spot test **(D)**

The multiplex PCR assay was performed using two primer sets to identify *E. faecalis* strains and the *VanA* gene. Of the 46 Enterococcus isolates, 13 were identified as Vancomycin-resistant *E. faecalis.* The 941 bp and 1030bp PCR products belong to *E. faecalis* and *vanA* genotype, respectively (Figure1A). The Minimum inhibitory concentration method of Vancomycin-Resistant for all *E. faecalis* was greater than 256 (≥256).

**Bacteriophage Isolation**

The double-layer agar test results showed that a new lytic phage from hospital sewage was isolated on the host vancomycin-resistant strains of *E*.* faecalis* V583. This phage formed clear zones with plaques ranging from 0.5 to 1 mm (Figure 1B).

**Electron Microscopy**

Transmission electron microscopy indicated that the phage V583 has an icosahedral head with a diameter of 122 ±0.3 nm and a tail length of 222 ±0.1 nm. Based on morphology, phage V583 belongs to the *Siphoviridea* family and *Caudovirales* order according to the ICTV classification system (Figure 1C). 

**Host Range Determination**

Lytic activity was checked for bacterial susceptibility to phage (Figure 1D). All 13 strains of Vancomycin-resistant *E. faecalis* were sensitive to phage V583; however, isolated phage could not infect any other enterococcal species (Table 2).

**Table 2:** Lytic spectra of phage V583 against the clinical isolates

Bacterial strain	Number of isolates	Plaque formation
VREfs	13	**100%**
*E. faecium*	34	**Not susceptible**
*E.* *gallinarum*	1	**Not susceptible**
JH-2-2	1	**Not susceptible**

**Inhibitory Effect of Phage on Biofilms**

**Fig. 2:**
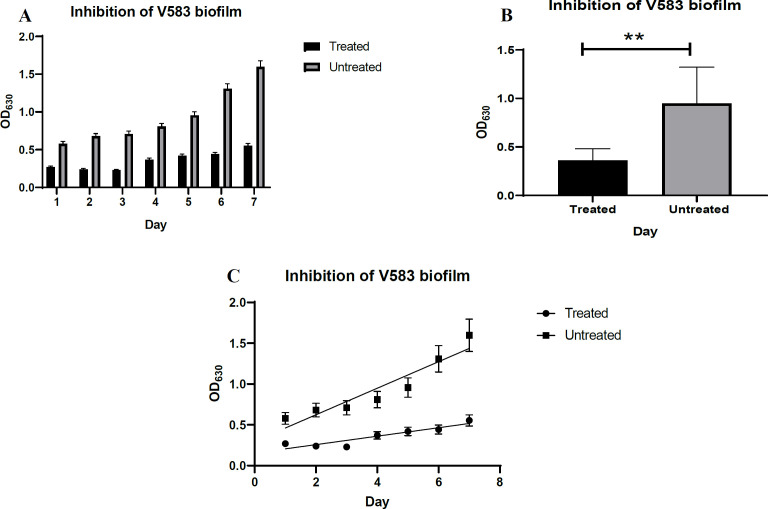
Inhibitory effect of phage V583 on biofilm. Comparison of mean data on biofilm inhibition with phage V583 at different time intervals (days 1-7) compared to the control at 630nm **(A)(C).** Unpaired T-test results for the comparison of data regarding biofilm inhibition by phage V583 over the course of 7 days compared to the control **(B).**

Figure 2A shows the results of biofilm inhibition by phage over 7 days. The efficacy of phage V583 against biofilm inhibition was significant for 1 to 7 days (*P* <0.05 on all days). Absorbance (OD 630) of treated biofilms was increased with the number of incubation days from day 4 to 7 compared to the previous days, and maximum absorbance was observed on day 7. The absorbance of biofilm on days 1 to 7 in phage-treated samples was significantly less than biofilm on days 1 to 7 in untreated samples. Furthermore, a thinner biofilm was formed in the treated samples. The results generally indicated a threefold inhibition of biofilm compared to control (*P*=0.001) (Figure 2B). As shown in Figure 2C, bacterial generation time in terms of absorption in the phage-treated biofilms increased compared to the untreated biofilms (13.38 and 4.26 minutes, respectively).

**Disruption Effect of Phage on Biofilms**

**Fig. 3:**
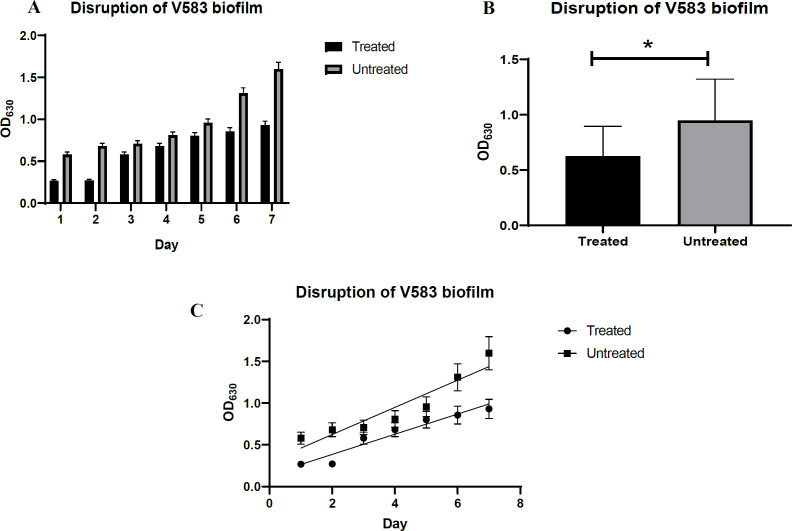
The disruptive effect of phage V583 on biofilms. Comparison of mean data on biofilm disruption with phage V583 at different time intervals (days 1-7) compared to the control at 630 nm **(A)(C).** Pair T-test results to compare the mean of biofilm disruption with phage V583 over the course of 7 days compared to the control **(B)**

Phage V583 destroyed 1 and 2-day biofilm but had no effect on biofilm on days 3 to 7 compared to the previous days (Figure 3A). In general, the absorbance of treated mature biofilm on days 1 to 7 was significantly lower than that of untreated mature biofilm on days 1 to 7. The results indicated that the biofilm was twofold degraded as the control (*P*=0.005) (Figure 3B). As shown in Figure 3C, the generation time of bacteria in terms of absorption in phage-treated biofilms increased compared to untreated biofilms (5.74 and 4.26 minutes, respectively). 

**Evaluation of Living Cells in the Biofilm**

**Fig. 4:**
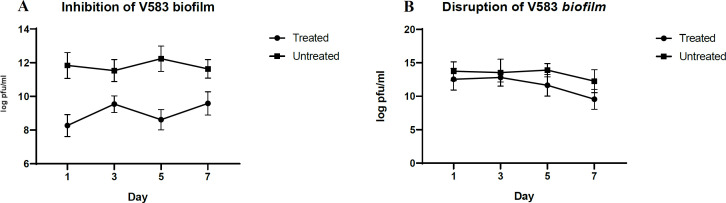
Log_10_ CFU of live *E. faecalis* in different days. Biofilm was treated with and without the phage under inhibition **(A)** and disruption **(B)** conditions

CFU assay was performed to quantify the number of viable cells within the biofilm. Figures 4(A) and (B) show the inhibitory and disruption effect of phage V583 on the treated biofilm. In disruption assay, the number of living cells in the treated biofilm decreases gradually compared to the control (untreated biofilm). This reduction was equal to 1.9 log, which can probably be due to the phage. In the inhibition assay, the number of living cells in the treated sample decreased during 7 days compared to the 2.8 log control.

## Discussion

Antimicrobial activities of many phages that infect strains of *E. faecalis* have been previously reported (25-27). In our study, using specific primers, among the enterococci samples collected from Shariati Hospital, 13 species of *E. faecalis* that were resistant to vancomycin with *vanA* genotype were identified. In a study conducted by Moghimbeigi *et al.* in 2018, the number of *E. faecalis* species was reported to be 68%, and most resistant strains (80% to 86%) showed the *vanA* genotype (28). In a 2007 study by Talebi *et al.* using Multiplex PCR, 48 isolates were resistant to vancomycin, and all carried the *vanA* gene, which is consistent with the results of our study (29). In this investigation, a specific lytic phage against vancomycin-resistant *E. faecalis* isolates was isolated from the wastewater of Shariati Hospital. In a study conducted by Leron Khalifa *et al.* in 2015, as in our study, the phage EFDG1 against *E. faecalis* was isolated from wastewater (30). Today, Lytic bacteriophages could become a new biocontrol of anti-biofilm agents, especially regarding multidrug-resistant biofilm infections. A biofilm is a population of cells growing on a surface and enclosed in a self-produced EPS Matrix (31). The nature of bacterial biofilm is resistant to killing by antimicrobials since MIC is higher than planktonic cells (32). The reason for this high tolerance to antibiotic therapy is not entirely discerned. It is thought that the existence of a slow growth rate of bacterial cells, persister cells, and exopolymeric matrix contributes to the resistance of biofilms (31, 33). The biofilm EPS matrix could also affect antimicrobial agents' efficacy due to diffusion limitations, deactivation, and drug binding (34). In a study conducted by Hanlon, G.W., *et al.* (2001), it was shown that phage would diffuse through alginate gels and *P. aeruginosa* EPS (35). In another study, the c2 Lactococcus phage movement occurs through biofilm water channels and cell clusters (36). Today, many studies have been performed using various bacteriophages to inhibit and destroy biofilms (37-39). These studies suggest that some of the most important barriers to biofilm control may be overcome by phage. In this case study, we demonstrated the isolation of a lytic phage that is more efficient against planktonic and biofilm cultures of *E. faecalis* V583 in vitro. The phage host range was evaluated on clinical isolates of *E. faecalis *and *E. faecium*, JH-2-2 (*E. faecalis* sensitive vancomycin) and *E. gallinarum*. According to our results, this phage can infect only *E. faecalis* (sensitive and resistant vancomycin). A narrow-spectrum is another advantage phage that enables it to eliminate pathogens without normal flora effects, unlike antibiotics. Electron microscopy showed that the isolated phage in our study belonged to the order *Caudovirales* and the family *Siphoviridae*. Previous study have isolated siphophages for *E. faecalis *(27). In our study, the effect of phage on the inhibition and degradation of *E. faecalis* biofilms was tested for 1 to 7 days. In inhibition assay, the effect of phage significantly reduced biofilm for up to 7 days, but in disruption effect, the phage's effectiveness was significant against 1-day-old to 2-day-old biofilms (Figures 2A and 3A). These results ensure the prosperity of phage therapy over antibiotic treatments in biofilms (40). Hanlon, G.W., *et al.* (2001) shows that biofilm's sensitivity to phage F116 does not decrease with increasing biofilm age, and this phage was active against biofilms that were 20 days old (35). In a study by Leron Khalifa, *et al.* (2015), EFDG1 was able to efficiently infect and kill planktonic and biofilm cultures of *E. faecalis* (26). Crystal violet staining showed a 5-fold decrease in biomass within 7 days compared to untreated samples (26). It is known that phage-biofilm interactions are a somewhat complicated process, and the possible mechanisms are not clear (41). In the initial attachment stage, secreted and surface proteins play essential roles and facilitate attachment and cell-to-cell signaling (42).The first bacteriophage infection stage is the adsorption of phage particles to the specific receptors on the bacterial cell's surface. Thus, phages bind to these receptors to inhibit biofilm formation (36). The high cell density of the biofilm can enhance phage replication (41, 43)*.* As shown in Figures. 2 and 3, the CV assay in biofilm inhibition and degradation experiments showed that phage-treated biofilms had less biomass than the untreated biofilms (*P*<0.05). The phage seemed to affect biofilm inhibition (*P*=0.0021) more than biofilm disruption (*P*=0.0332). This observation may be due to biofilm maturation, thick extracellular matrix layer, lower permeability to phage, reduced viable cell number, and slow bacterial growth (31, 33). Previous studies have shown that there is no stable pattern for the number of living and dead cells in the biofilm on different days, which is probably due to the complex effects of mortality on the natural life cycle of bacteria and the environment within the biofilm (44). The results of the CFU assay support the reduction of cell growth in the presence of the phage. This decrease in inhibition and degradation experiments was 2.8 and 1.9 logs compared to controls, respectively. For example a study conducted in 2019 by Mor Shlezinger, *et al.* indicated that phage against *E. faecalis* biofilm causes a 95% reduction in the number of living cells and an 88% reduction in bacterial biomass (44). In conclusion, phage V583 possesses characteristics that can be considered as a supplementary or alternative strategy to conventional antibiotic treatment precisely in the case of biofilm. 

## Conclusion

Treatment of vancomycin-resistant enterococcal faecalis infections has become a challenge today. Therefore, finding alternatives or supplements for antibiotics is a necessity. The unique properties of phages among antibiotic-resistant bacteria have made them a promising tool. In this study, we isolated *E. faecalis* specific lytic phage and investigated the effect of phage on biofilm in the presence and absence of phage. As the results showed, phage v583 effectively removed biofilms in both inhibition and degradation methods, which emphasizes the importance of using this phage in the control of biofilms resistant to this bacterium.

## Conflict of Interest

The authors declared no conflict of interests.

## Funding

None.
